# Diffuse Gastric Ganglioneuromatosis: Novel Presentation of *PTEN* Hamartoma Syndrome—Case Report and Review of Gastric Ganglioneuromatous Proliferations and a Novel *PTEN* Gene Mutation

**DOI:** 10.1155/2018/4319818

**Published:** 2018-03-25

**Authors:** Alexander J. Williams, Emily S. Doherty, Michael H. Hart, Douglas J. Grider

**Affiliations:** ^1^Carilion Clinic, Roanoke, VA, USA; ^2^Carilion Clinic Children's Hospital, Roanoke, VA, USA; ^3^Dominion Pathology Associates, Roanoke, VA, USA; ^4^Virginia Tech Carilion School of Medicine, Roanoke, VA, USA

## Abstract

Gastrointestinal ganglioneuromatous proliferations are rare, most often found in the colon, and are three types: polypoid ganglioneuromas, ganglioneuromatous polyposis, and diffuse ganglioneuromatosis. We present a case of diffuse ganglioneuromatosis in the posterior gastric wall in a nine-year-old female. To our knowledge, this is the first reported case of diffuse ganglioneuromatosis located in the stomach. Only six cases of gastric ganglioneuromatous proliferations have previously been reported, two in English and none were diffuse ganglioneuromatosis. A diagnosis of diffuse ganglioneuromatosis is relevant for patient care because, unlike sporadic polypoid ganglioneuromas or ganglioneuromatous polyposis, most are syndromic. Diffuse ganglioneuromatosis is commonly associated with neurofibromatosis type 1, multiple endocrine neoplasia type 2b, and Cowden Syndrome, one of the phenotypes of *PTEN* hamartoma tumor syndrome. The patient had the noted gastric diffuse ganglioneuromatosis, as well as other major and minor criteria for Cowden syndrome. Genetic testing revealed a novel frameshift mutation in the *PTEN* gene in the patient, her father, paternal aunt, and the aunt's son who is a paternal first cousin of the patient.

## 1. Introduction

Gastrointestinal ganglioneuromas are rare tumors, most often found in the descending colon, rectum and occasionally the appendix. They are of three types: polypoid ganglioneuromas, ganglioneuromatous polyposis, and diffuse ganglioneuromatosis [1, 2]. Polypoid ganglioneuromas are usually single polyps that are sporadic and indolent. Ganglioneuromatosis polyposis, as the name implies, consists of many polyps, while diffuse transmural ganglioneuromas have a high association with neurofibromatosis type 1 (NF1), multiple endocrine neoplasia type 2b (MEN 2b), and *PTEN* hamartoma syndrome [3] (OMIM 601728). Less well-known associations include Hirschsprung's disease, tuberous sclerosis, familial adenomatous polyposis, and juvenile polyposis [4, 5].

Our case is unique because it is one of only six previously reported ganglioneuromatous proliferations found in the stomach and is the first case of diffuse ganglioneuromatosis [6–9]. This case also identifies a previously unreported mutation in the *PTEN* gene consistent with the *PTEN*-related findings observed clinically in our patient, her father, paternal aunt, and the aunt's son who is a paternal cousin of the patient.

## 2. Case Presentation

**Figure 1:**
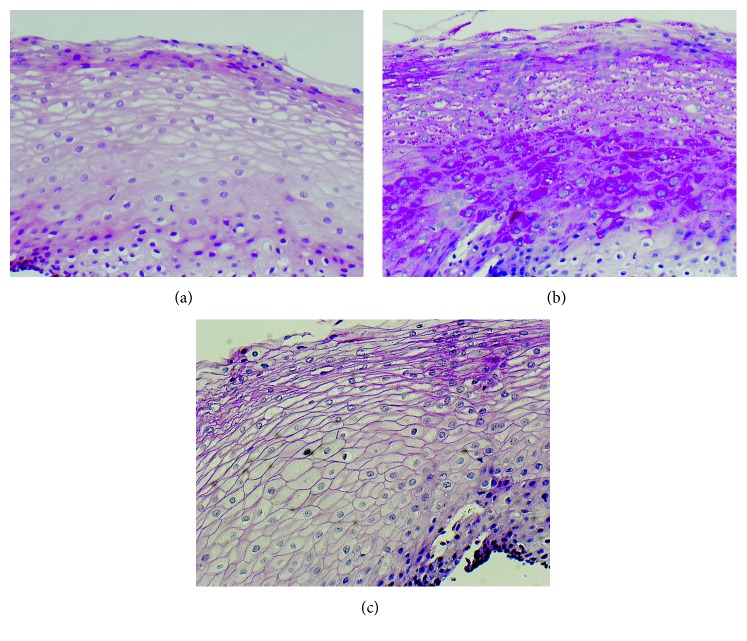
(a) Esophageal squamous mucosa with upper keratinocytes showing “cleared out” cytoplasms, characteristic for glycogenic acanthosis, and a rare eosinophil, consistent with minimal reflux (H&E 20x). (b) PAS without diastase digestion showing granular magenta positivity in keratinocytic cytoplasms of the upper half of the squamous mucosa, typical for glycogenic acanthosis (PAS 20X). (c) PAS with diastase digestion showing absence of the positive magenta granularity in the keratinocytic cytoplasms of the upper half of the squamous mucosa, confirming glycogenic acanthosis (PASD 20x).

A nine-year-old Caucasian female presented with symptoms of reflux and postprandial gagging, dysphagia, epigastric pain, and fecal withholding. Her medical history was significant for prematurity, born at twenty-five weeks gestation and related conditions of prematurity including retinopathy, anemia, and lung disease. She was born with a mild ventricular septal defect and a patent ductus arteriosus that failed to close with two trials of indomethacin and required surgical ligation. After birth, she spent four months in the neonatal intensive care unit, requiring supplemental oxygen and placement of a percutaneous endoscopic gastrostomy tube for feeding. She was diagnosed with esophageal glycogenic acanthosis (Figures 1(a)–1(c)) found on upper endoscopy performed at age twenty-two months for symptoms of reflux and feeding difficulties. She had retinopathy of prematurity that was treated with laser surgery and strabismus that was treated with botulinum injections. Macrocephaly, global developmental delays, and a limited attention span were present in early childhood. MRI of her brain at age 7 years showed signs of mild periventricular leukomalacia and no other abnormalities.

On exam, the patient was developmentally delayed for her age, and her head circumference was 56.4 cm, which is macrocephalic for age (98th percentile for head circumference in a 9-year-old girl is 55 cm) [10]. Skin exam showed well-healed scars from prior surgeries but no lipomas or other mucocutaneous features of Cowden syndrome. The remainder of the examination was unremarkable.

**Figure 2:**
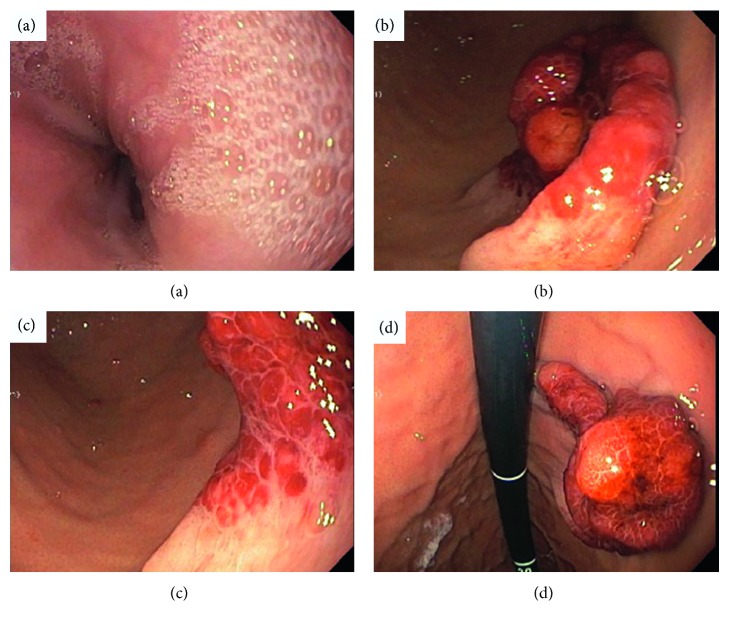
Upper endoscopy: (a) normal distal esophagus; and (b–d) gastric mass with pedicle.

**Figure 3:**
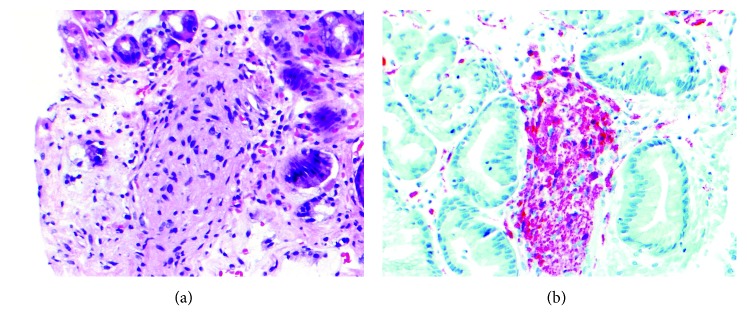
(a) Gastric biopsy showing spindled cells in fascicles with slightly atypical nuclei in the mucosal lamina propria (H&E 20X). (b) Gastric biopsy with spindle cells in the lamina propria showing strong S100 protein positivity (S100 protein 20x).

**Figure 4:**
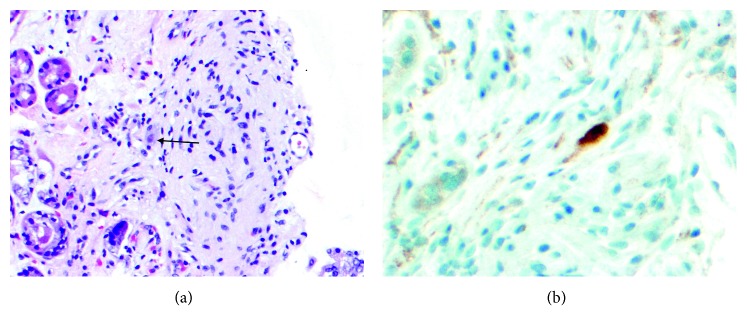
(a) Gastric biopsy with only ganglion cell amongst spindle cells, tip of black arrow (H&E 20X). (b) Neuron specific enolase (NSE) marking the single ganglion cell in gastric biopsy (NSE 40x).

Due to her clinical symptoms, upper endoscopy was performed, and a variegated fungating mass with very prominent lymphoreticular nodularity and friability was found along the medial aspect of the lesser curvature of the stomach at the juncture of the body with the antrum. The size of the mass was difficult to estimate on endoscopy but appeared to be 2 cm in width and 3 cm in length on a pedicle that was about 1.5 cm at the base (Figures 2(a)–2(d)). Biopsies of the mass were obtained at several levels, including the base and tip. The remainder of the stomach, esophagus, and upper duodenum were also sampled showing reactive gastropathy, mild reflux changes, and mild active duodenitis, respectively. Microscopy of the biopsied gastric mass was interpreted to be a ganglioneuromatous proliferation with small foci in the lamina propria of spindled cells in fascicles with slightly atypical nuclei, positive on S100 protein and SOX-10 immunohistochemically stained tissue sections (Figures 3(a) and 3(b) a single, well-formed ganglion cell was noted in one of the areas of spindle cells, positive for neuron specific enolase (NSE) by immunohistochemistry (Figures 4(a) and 4(b)). Ki-67 was negative in the spindle cells, supporting a low proliferative index. The spindled cells were negative for CD34 and CD117, helping exclude gastrointestinal stromal tumor. Epithelial membrane antigen (EMA) was negative, suggesting against a perineuroma. Keratin AE1/AE3, SMA, and melan-A were all negative, suggesting against a spindled carcinoma, melanoma, and leiomyomatous proliferation. GFAP was negative, as sometimes can be seen in Schwann cells and ganglion cells [11].

**Figure 5:**
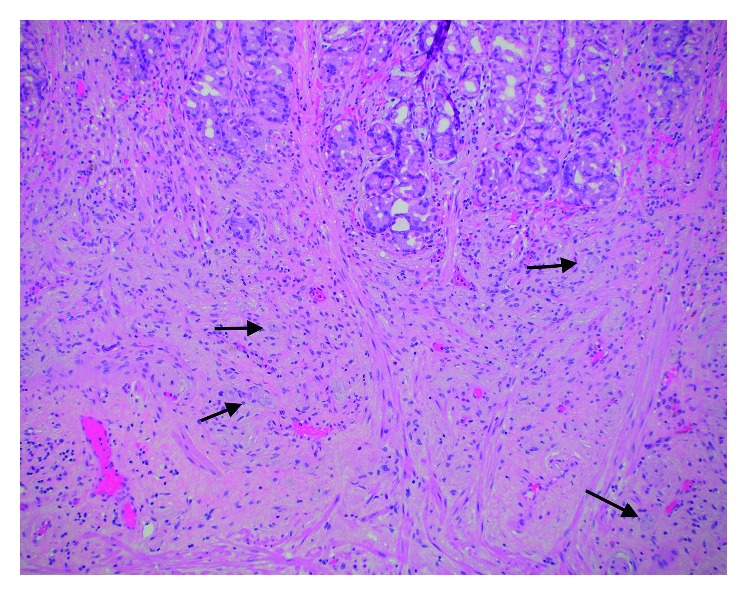
Partial gastrectomy specimen with ganglioneuromatous proliferation extending from the submucosa into the lamina propria splaying oxyntic mucosal glands. A few of the ganglion cells are annotated with arrows (H&E 4x).

**Figure 6:**
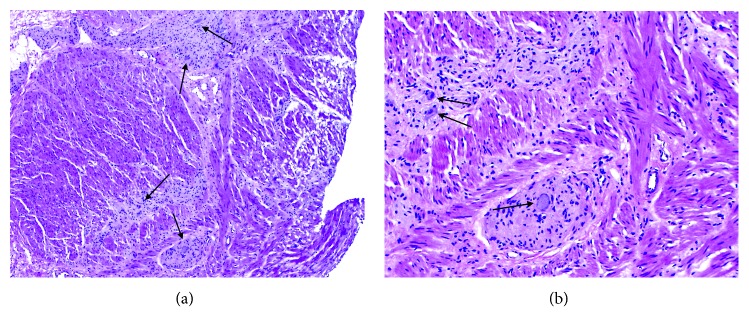
(a) Frozen section taken at time of partial gastrectomy showing muscularis propria with a myenteric ganglioneuromatous proliferation. Arrows mark some of the ganglion cells (H&E 4x). (b) Higher power image of the gastric resection at frozen section confirming a myenteric ganglioneuromatous proliferation. Ganglion cells marked with arrows (H&E 10x).

Three weeks later, a partial gastrectomy was undertaken to completely remove the mass, confirmed as a ganglioneuromatous proliferation of the diffuse ganglioneuromatosis type (Figures 5 and 6(a) and 6(b)). No polygonal or columnar cells typically seen in paragangliomas were noted, and the presence of ganglion cells excluded schwannoma and neurofibroma. Thus, the diagnosis of diffuse ganglioneuromatosis was confirmed. The patient's symptoms of reflux, postprandial gagging, dysphagia, and epigastric pain resolved following surgery.

## 3. Family History

The patient's father had a prior history of macrocephaly and a large benign tumor removed from his left flank at age 17 years. Past medical history was also significant for diabetes mellitus and testicular lipoma. He presented at age 29 years with a symptomatic multinodular goiter. Thyroidectomy was performed. The pathology of the gland revealed multifocal papillary thyroid cancer and nodular dysplasia with the largest nodule being 2.4 cm. He had one lymph node sampled that was negative for metastasis, and he was given radioactive iodine therapy for definitive treatment. His primary care provider diagnosed him with “multiple hamartoma syndrome.” His examination at age 31 years was notable for macrocephaly, subcutaneous lipomas, penile freckling, and many axillary skin tags. The rest of the patient's family history was remarkable for a paternal aunt that underwent partial gastric resection at age nineteen for what was diagnosed as a hamartomatous polyp, unspecified, and thyroid goiter that was treated with thyroidectomy. The paternal aunt had a son with symptomatic hydrocephalus that was shunted at age 2 years, macrocephaly, type 1 Chiari malformation, and penile freckling. The patient's paternal grandfather died at age twenty-seven years from renal and lung cancer.

## 4. Discussion

The differential diagnosis of spindle cell tumors includes gastrointestinal stromal tumors (GIST), schwannoma, leiomyoma, neurofibroma, gangliocytic paraganglioma, and ganglioneuroma [1, 12]. The presence of ganglion cells and negative staining for CD117 and CD34 in the spindled cells excludes the diagnosis of GIST. Schwann cell proliferations of the stomach are most likely to be a schwannoma or neurofibroma; however, both are excluded by the presence of ganglion cells. Negative SMA stain excludes leiomyoma. Lastly, gangliocytic paraganglioma is a triphasic tumor of epithelioid, ganglion, and spindle cells usually occurring in the ampulla of Vater. Gangliocytic paraganglioma is excluded because no polygonal or columnar epithelioid cells, usually keratin positive, are found, and the keratin AE1/AE3 is negative in the mucosal biopsy specimen.

Ganglioneuromas are rare tumors of the GI tract composed of ganglion cells, nerve fibers, and supporting cells [13]. Polypoid ganglioneuromas are most often small, sessile, or pedunculated polyps that grossly resemble juvenile polyps, adenomas, or hyperplastic polyps. Microscopically polypoid ganglioneuromas can appear as collections of spindle cells in a fibrillary matrix, irregular groups, and nests of ganglion cells in the lamina propria. They can also appear as a nodular mucosal and submucosal ganglion and spindle cell proliferation that suggests a neurofibroma or as nodular mucosal proliferations of clustered ganglion cells admixed with varying amounts of spindle cells without any significant disarray of the mucosal architecture [14]. There have been some cases of polypoid ganglioneuromas reported in Cowden syndrome [14, 15] as found on surveillance endoscopy in this patient.

Ganglioneuromatosis polyposis is distinguished by numerous sessile or pedunculated mucosal and/or submucosal lesions showing greater variability in neural, supportive, and ganglion cell content with demarcation compared to polypoid ganglioneuromas. Diffuse ganglioneuromatosis is a poorly demarcated, nodular, and diffuse intramural or transmural proliferation of ganglioneuromatous tissue that diffusely involves the enteric, most often myenteric, nerve plexuses. The histological growth pattern varies from fusiform, hyperplastic expansions of the myenteric plexus to confluent, irregular, transmural ganglioneuromatous proliferations that distort the myenteric plexus and infiltrates the adjacent bowel wall.

A ganglioneuroma of the stomach is extremely unusual, with only six cases previously reported. Our patient's mass had the endoscopic appearance of a juvenile polyp but the histology of a ganglioneuromatous proliferation. Polypoid ganglioneuroma is not favored due to the presence of diffuse transmural involvement of the gastric wall, favoring diffuse ganglioneuromatosis. Ganglioneuromatous polyposis is not favored because polyposis is not present. Diffuse ganglioneuromatosis of the GI tract is associated with other tumors and syndromes, including *PTEN* hamartoma syndrome, MEN 2b, NF1 (von Recklinghausen's disease), and neurogenic sarcoma [16].

## 5. Discussion of Genetic Testing and *PTEN*-Related Disorders

**Figure 7:**
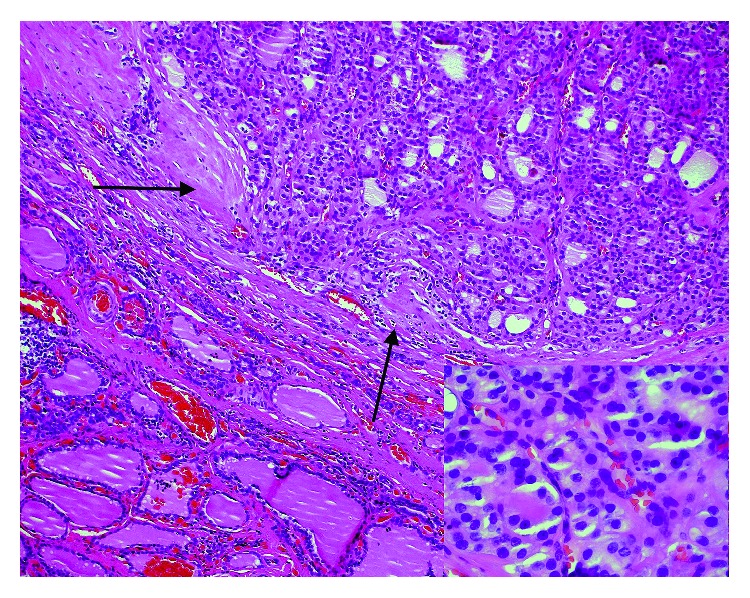
Minimally invasive follicular thyroid carcinoma showing invasion completely through the fibrous capsule, arrows, but not into the thyroid parenchyma (H&E 10X); lower right inset shows nuclear features of follicular carcinoma; absent are the nuclear pseudoinclusions and nuclear grooves expected in the follicular variant of papillary thyroid carcinoma (H&E 60x).

*PTEN* gene sequencing was performed on the patient and her father in a CLIA-approved commercial laboratory (GeneDx, Gaithersburg, MD). A c.271_272delGAinsTT mutation in the *PTEN* gene was found in both individuals. The mutation is expected to cause a frameshift, in which the glutamic acid at codon 91 is changed to phenylalanine, and a premature stop codon is created at position 4 of the new reading frame, denoted p.Glu91PhefsX4. This mutation is predicted to cause loss of normal protein function either through protein truncation or nonsense-mediated mRNA decay. Although this mutation has not been previously reported to our knowledge, its presence is consistent with the clinical diagnosis of Cowden syndrome in this patient and her father. Both individuals were educated by a clinical geneticist regarding the diagnosis, and an appropriate vigilant cancer surveillance program [17] was tailored to the patient and her father. On the patient's initial thyroid ultrasound, a thyroid nodule was discovered. The patient's parents elected a thyroidectomy instead of clinical surveillance. A total thyroidectomy was performed that revealed both a minimally invasive follicular carcinoma (Figure 7) and lymphocytic thyroiditis. Yearly surveillance upper endoscopy and lower endoscopy studies showed a colonic polypoid ganglioneuroma at 30 cm at age 10 and two additional distal colonic polypoid ganglioneuromas at age 12. The patient's paternal aunt and her son also had genetic testing, and were found to carry the same *PTEN* mutation. They also received genetic counseling and were started on a cancer surveillance program.

The phosphatase and tensin homolog (*PTEN*) gene, at cytogenetic location 10q23.31, negatively regulates signaling pathways that are critical for cell proliferation, cell cycle progression, and apoptosis. Loss of function of this gene either through inherited or sporadic mutations contributes to oncogenesis, as it is considered to be a tumor suppressor gene [18]. Germline mutations of *PTEN* have been described in a variety of rare and clinically underrecognized syndromes, collectively known as *PTEN* hamartoma tumor syndrome (OMIM 601728). The phenotypic spectrum of *PTEN*-related syndromes includes two allelic disorders linked to mutations in the *PTEN* gene: Cowden syndrome and Bannayan–Riley–Ruvalcaba syndrome [19]. The relation of Proteus syndrome and Proteus-like syndrome with *PTEN* mutations is controversial [20].
